# Seroprevalence of *Toxoplasma gondii* among healthy blood donors in two locations in Tunisia and associated risk factors

**DOI:** 10.1051/parasite/2020049

**Published:** 2020-09-21

**Authors:** Arwa Lachkhem, Ibtissem Lahmar, Lokman Galal, Oussama Babba, Habib Mezhoud, Mohssen Hassine, Ahmed Lachkhem, Marie-Laure Dardé, Aurélien Mercier, Hamouda Babba

**Affiliations:** 1 Laboratoire de Parasitologie–Mycologie Médicale et Moléculaire, Département de Biologie Clinique B, Faculté de Pharmacie de Monastir, Université de Monastir 5000 Monastir Tunisia; 2 INSERM, Université Limoges, CHU Limoges, IRD, U1094 Neuroépidémiologie Tropicale, Institut d’Epidémiologie et de Neurologie Tropicale, GEIST 87000 Limoges France; 3 Centre de Maternité et de Néonatologie de Monastir 5000 Monastir Tunisia; 4 Hématologie – Banque du Sang de Monastir 5000 Monastir Tunisia; 5 Centre de Transfusion Sanguine de Gafsa 2100 Gafsa Tunisia; 6 Centre National de Référence (CNR) Toxoplasmose/Toxoplasma Biological Center (BRC), Centre Hospitalier-Universitaire Dupuytren 87000 Limoges France

**Keywords:** *Toxoplasma gondii*, Blood donors, Seroprevalence, Risk factors, Tunisia

## Abstract

*Toxoplasma gondii* is a protozoan parasite that can be transmitted to humans through a variety of routes including blood transfusion. This study aimed to investigate the seroprevalence of *T*. *gondii* infection and associated risk factors in healthy blood donors in Tunisia. A total of 800 healthy blood donors from two blood centers in south and coastal Tunisia were analyzed for anti-*T. gondii* IgG and IgM antibodies by indirect immunofluorescence assay (IFA) and enzyme-linked immunoassays (ELISA), respectively. Structured questionnaires were used to gather information on risk factors for *T*. *gondii* infection during collection. The overall seroprevalence was 44.4% of which 352 (44%) and 3 (0.4%) were positive for IgG and both IgG and IgM anti-*T. gondii* antibodies, respectively. Multivariate analysis showed that *T. gondii* seropositivity was significantly associated with the birth place (adjusted odds ratio [OR] = 2.72; 95% confidence interval [CI]: 1.49–4.94) and the age of the donors (adjusted OR = 4.98; 95% CI: 1.50–16.58) which are independent risk factors. In addition, the variables of hand washing before eating (adjusted OR = 0.52; 95% CI: 0.37–0.74) and living in an urban environment (adjusted OR = 0.30; 95% CI: 0.13–0.71) are two protective factors. This study provided the first data on the seroprevalence and epidemiology of *T. gondii* infection in healthy blood donors in Tunisia.

## Abstract (fr)

*Toxoplasma gondii* est un parasite protozoaire qui peut être transmis à l’homme par diverses voies, dont la transfusion sanguine. Cette étude vise à étudier la séroprévalence de l’infection à *T. gondii* et les facteurs de risque associés chez les donneurs de sang sains en Tunisie. Au total, huit cents donneurs de sang sains de deux centres de transfusion sanguine du sud et de la côte tunisienne ont été analysés respectivement pour la recherche des anticorps IgG et IgM anti-*T. gondii* par immunofluorescence indirecte (IFA) et par dosage immuno-enzymatique (ELISA). Des questionnaires structurés ont été utilisés pour recueillir des informations sur les facteurs de risque d’infection à *T. gondii* pendant la collecte. La séroprévalence globale était de 44,4 % dont 352 (44 %) et 3 (0,4 %) étaient respectivement positifs pour les anticorps IgG et IgG/IgM anti-*T. gondii*. Une analyse multivariée a montré que la séropositivité à *T. gondii* était significativement associée au lieu de naissance (rapport de côtes ajusté [OR] = 2,72 ; intervalle de confiance à 95 % [IC] : 1,49–4,94) et à l’âge des donneurs (OR ajusté = 4,98 ; IC 95 % : 1,50–16,58) qui sont des facteurs de risque indépendants. De plus, le lavage des mains avant de manger (OR ajusté = 0,52 ; IC 95 % : 0,37–0,74) et vivre dans un milieu urbain (OR ajusté = 0,30 ; IC 95 % : 0,13–0,71) sont deux facteurs de protection. Cette étude a fourni les premières données sur la séroprévalence et l’épidémiologie de l’infection à *T. gondii* chez les donneurs de sang sains en Tunisie.

## Introduction

Toxoplasmosis is a widespread cosmopolitan zoonosis due to a protozoan parasite, *Toxoplasma gondii*, which affects one-third of the world’s population [14, 31]. Acquired toxoplasmosis is usually benign and asymptomatic among immunocompetent people. However, severe diseases and complications can occur in immunocompromized persons and congenitally infected children [19, 32, 39]. In fact, the clinical manifestations of toxoplasmosis are affected by the genotype of the parasite, which can be serious during infection with a strain of atypical genotype [18, 20, 23].

The seroprevalence of *Toxoplasma* infection in humans varies widely between countries (10–80%) and sometimes within a country, depending on social and cultural habits, geographic factors, climate, and transmission routes [37].

Human infection can be horizontal by eating undercooked or raw meat containing tissue cysts, ingesting tachyzoites in milk, or oocysts in water, food or soil contaminated with infected cat’s feces. *Toxoplasma* can also be transmitted vertically to the fetus through the placenta during pregnancy. In addition, blood transfusion could be another route of transmission of *T. gondii* infection that could cause serious problems in immunocompromized and multitransfused persons [27]. Seropositive blood donors, particularly those who are in the acute stage of infection, could in rare cases play a role in transmission [13].

It has been demonstrated that *Toxoplasma* tachyzoites can survive in stored blood for several weeks [25], which increases the risk of transmission by blood transfusion [37].

In Tunisia, a few studies have focused on the seroprevalence of *Toxoplasma* infection in the general population [4] and in pregnant women [11, 34]. However, the seroprevalence in healthy blood donors remains totally uninvestigated. Therefore, the objective of the current study was to assess the seroprevalence of IgG and IgM anti-*T. gondii* antibodies and identify the associated risk factors among healthy blood donors in South and Coastal Tunisia.

## Materials and methods

### Ethical considerations

Our study was conducted according to the tenets of the Declaration of Helsinki. This study was approved by the Ethics Committee of the Monastir Medical Faculty, Tunisia (IORG 0009738N°21/OMB 0990-0279). All participants were informed about the purpose and procedures of the survey. Sera were collected with the written consent of the volunteers. For uneducated donors, informed consent was completed by legally authorized representatives.

### Study design and sample size

A prospective cross-sectional study was carried out in two Tunisian centers of blood transfusion. The first is located in the city of Monastir in Central-Eastern Tunisia and covers an area of 1024 km^2^ and has 548,828 inhabitants (Tunisian National Institute of Statistics, 2014). Administratively, the governorate is divided into 13 delegations. It is a coastal city of the Sahel that has a semi-arid climate, and the winter is relatively cool and quite wet. The city is well known for tourism. The average annual temperature and precipitation are 18.1 °C and 328 mm, respectively (https://www.Climate-data.org).

**Figure 1:**
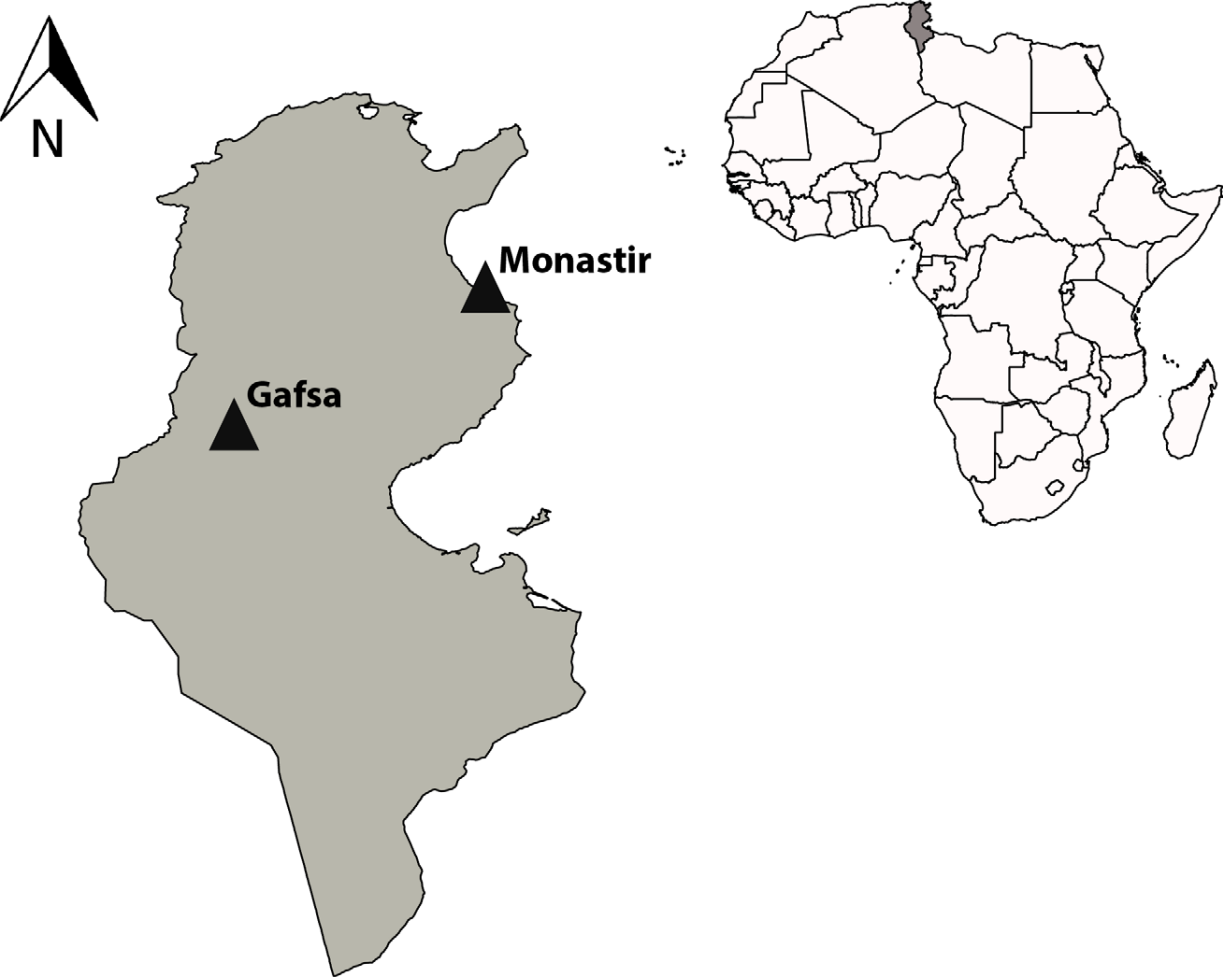
Location of the two study regions on a map of Tunisia.

The second center is located in the city of Gafsa in the South-West of Tunisia and covers an area of 8990 km^2^ and has 337,331 inhabitants (Tunisian National Institute of Statistics, 2014). It consists of thirteen delegations. It has an arid climate, with the dry season extending almost to the whole year. Gafsa is known for its phosphate deposits, but the city is rather poor and does not benefit from phosphate revenues. On average, the temperature is 19.2 °C, and rain is practically non-existent (161 mm) (https://www.Climate-data.org) (Fig. 1).

In the absence of previous studies on toxoplasmosis among blood donors in Tunisia, we estimated the sample size using the following formula: *n* = *p* (1 − *p*) *Z* ^2^/*d* ^2^ where: (*n*) required number of blood donors to be sampled, (*p*) 50% expected prevalence and a 95% confidence interval (*Z* = 1.96) with a 5% desired absolute precision (*d*) [38], According to this calculation, the required sample size for each region was *n* = 400.

### Sample collection and participants

In total, 800 serum samples were collected from healthy blood donors who were referred to the two blood centers (Gafsa, *n* = 400 and Monastir, *n* = 400) from January 2017 to January 2018. None of the blood donors was seropositive for the routinely tested infections: immunodeficiency virus (HIV), hepatitis C virus (HCV), hepatitis B virus surface antigen (HBs Ag) and *Treponema pallidum*.

About 5 mL of venous blood were collected aseptically from each of the study participants. Sera were separated from whole blood by centrifugation at 3000 rpm for 10 min, then labeled and kept at −20 °C until use.

### Survey

The applied questionnaire covered socio-demographic information including birth place, age, gender, residence area, education level, level of income (a donor is considered to have a low income when s/he spends less than 1085 dinars/year according to the Tunisian National Institute of Statistics, 2015; equivalent in Euros: 334; in dollars: 396), marital status, occupation and (ABO/RhD) blood group. Moreover, possible risk factors such as contact with cats, consumption of unwashed raw vegetables and fruits, consumption of undercooked meat, gardening or practice of agriculture and hand washing before eating, had been selected based on the literature.

### Serologic tests

Immunofluorescence assay (IFA) was used for the detection of IgG anti-*T. gondii* antibodies. IFA was performed as described with some modifications [33]. Briefly, slides coated with *T. gondii* RH strain tachyzoites derived from the peritoneal exudate of Swiss mice, probed with sera at serial dilutions of 1:20, 1:40, 1:80, 1:160 and 1:320, were prepared from each sample to be tested. Then, anti-human IgG conjugate (Fluoline G, bioMérieux^®^) was used at a dilution of 1:100 in phosphate-buffered saline with 1% Evans Blue (Sigma). A positive control marketed at a known concentration of 340 IU/mL (ToxotrolF, bioMérieux), a negative control, and a conjugated control were included in each analysis.

All slides were examined with a fluorescence microscope (LEICA DM 1000 LED). The positivity threshold was 1:20 corresponding to 8 IU/mL.

For the detection of *T. gondii* IgM antibodies, all the serum samples were tested using the commercially available Enzyme-Linked Immunosorbent Assay (ELISA) Kit (Patelia™ TOXO IgM, Bio-Rad, France) following the manufacturer’s instructions.

Positive results for IgM were defined as index values of ≥1.0, equivocal results ranged from index values of ≥0.8 – <1.0, and negative results were defined as index values of <0.8. IgG avidity was analyzed using a VIDAS^®^ TOXO IgG Avidity Kit (bioMérieux, France).

### Statistical analyses

Results were analyzed with XLSTAT, version 2017 (Windows). The risk factor analysis was conducted in two steps: univariable and multivariable analyses. In the univariable analysis, each independent variable was cross-correlated with the dependent variable (seropositivity). Multivariate analysis was used to assess the association between characteristics of subjects and *T. gondii* infection. Noncollinear qualitative variables were included in the multivariate analysis if they had a *p* value ≤ 0.25 in the univariate analysis. We integrated the variable “blood group” in the multivariate model as a random factor. The model was constructed using the backward stepwise exclusion method. Adjusted odds ratios (ORs) and 95% confidence intervals (CIs) were calculated using multiple logistic regression. A *p* value < 0.05 was considered statistically significant. The Hosmer–Lemeshow quality test was used to assess the adequacy of the model [5]. The reliability of the model was evaluated by the characteristic operating curve of the receiver. The interactions deemed relevant between the variables of the final model were tested.

## Results

### Seroprevalence of anti-*T. gondii* antibodies

Among 800 blood donors, 355 were seropositive for anti-*T. gondii* antibodies (44.4%, 95% confidence interval CI: 40.93–47.82). Of these, 352 cases (44.0%) tested positive only for IgG, suggesting a chronic infection profile. Three cases (0.4%) tested positive for both IgG and IgM, suggesting that more recent infection cannot be excluded. The determination of the IgG avidity index confirms an infection of more than four months.

Of the 355 seropositive blood donors, 151 (42.5%) had a titer of anti-*T. gondii* IgG antibodies of 8 IU/mL, 114 (32.1%) had a titer of 16 IU/mL, 77 (21.7%) 32 IU/mL, and 13 (3.7%) 64 IU/mL.

In terms of geographical region, seroprevalence of anti*-T. gondii* in the Monastir region at 48.0% (95% CI: 43.10–52.90) was significantly different from that of the Gafsa region 40.7% (95% CI: 35.93–45.57) (*p* < 0.05).

### Socio-demographic characteristics and other risk factors associated with seropositivity

The analysis of socio-demographic characteristics shows that most participants 79.5% (636/800) were males. The average age of the participants was 35 years (ranging from 18 to 62 years); the highest seroprevalence of infection was 58.3% (95% CI: 52.10–64.57) in the age group 26–35 years (*p* < 0.0001), while the lowest frequency 34.1% (95% CI: 28.68–39.60) was observed in the age group of 18–25 years.

Donors in rural areas were more frequently seropositive 54.0% (95% CI: 44.78–63.33) than those living in urban areas 42.8% (95% CI: 39.12–46.51), *p* = 0.028.

In addition, there was a significant difference according to marital status with a seroprevalence of 48.2% (95% CI: 43.07–53.35) in married versus 41.2% (95% CI: 36.58–45.80) in single individuals, *p* = 0.047 and to the occupation, specifically other business 50.2% (95% CI: 43.66–56.79) and employed 48.1% (95% CI: 41.70–54.47) compared to students 38.0% (95% CI: 30.88–45.10) and unemployed 38.04% (95% CI: 30.58–45.49), *p* < 0.05. In the same context, we observed that there are jobs that are strongly associated with the infection such as medical staff (50%), civil (52.3%), farmer (76.9%), butcher (100%), driver (70%), fisherman (80%) and tourist guide (100%). Demographic characteristics of the blood donors are shown in Table 1.

**Table 1:** Results of univariate and multivariate logistic regression analyses of *Toxoplasma gondii* seropositivity of blood donors in Tunisia.

Risk factors	*N* (positive)	Seroprevalence in % (95% CI)	Univariable		Multivariable	
			OR (95% CI)	*p*-value	OR (95% CI)	*p*-value
Region						
Gafsa	400 (163)	40.75 (35.93–45.57)				
Monastir	400 (192)	48.00 (43.10–52.90)	1.34 (1.01–1.77)	0.0390	1.72 (1.26–2.35)	0.0010
Gender						
Male	636 (282)	44.34 (40.48–48.20)				
Female	164 (73)	44.51 (36.91–52.12)	1.00 (0.71–1.42)	0.968		
Age (years)						
18–25	290 (99)	34.14 (28.68–39.60)				
26–35	240 (140)	58.33 (52.10–64.57)	2.70 (1.90–3.84)	<0.0001	2.79 (1.93–4.04)	<0.0001
36–45	175 (63)	36.00 (28.89–43.11)	1.08 (0.73–1.61)	0.6831	1.04 (0.69–1.58)	0.8340
>45	95 (53)	55.79 (45.80–65.78)	2.43 (1.52–3.90)	0.0002	0.60 (0.39–0.93)	0.0003
Residence area						
Rural	111 (60)	54.05 (44.78–63.33)				
Urban	689 (295)	42.82 (39.12–46.51)	0.64 (0.42–0.95)	0.0280	0.30 (0.13–0.71)	0.0064
Blood group						
A	216 (99)	45.83 (39.19–52.48)				
AB	38 (22)	57.89 (42.20–73.59)	1.62 (0.81–3.26)	0.1724	1.90 (0.91–3.93)	0.086
B	101 (50)	49.50 (39.75–59.26)	1.16 (0.72–1.86)	0.5418	1.14 (0.69–1.88)	0.600
O	445 (184)	41.35 (36.77–45.92)	0.83 (0.60–1.16)	0.2746	0.95 (0.67–1.35)	0.797
RhD						
Positive	725 (321)	44.28 (40.66–47.89)				
Negative	75 (34)	45.33 (34.07–56.60)	1.04 (0.64–1.68)	0.861		
Education level						
Uneducated	18 (10)	55.56 (32.60–78.51)				
Primary	136 (68)	50.0 (41.60–58.40)	0.80 (0.30–2.15)	0.6582	0.63 (0.22–1.85)	0.403
Secondary	321 (146)	45.48 (40.04–50.93)	0.67 (0.26–1.73)	0.4068	0.50 (0.18–1.43)	0.198
University	325 (131)	40.31 (34.97–45.64)	0.54 (0.21–1.40)	0.2066	0.51 (0.18–1.50)	0.224
Level of income						
Low	71 (35)	49.30 (37.67–60.93)				
Intermediate and high	729 (320)	43.90 (40.29–47.50)	1.24 (0.76–2.02)	0.383		
Consumption of undercooked meat						
No	698 (298)	42.69 (39.02–46.36)				
Yes	102 (57)	55.88 (46.25–65.52)	1.70 (1.12–2.58)	0.0130	1.33 (0.85–2.10)	0.242
Consumption of unwashed raw vegetables and fruits						
No	361 (150)	41.55 (36.47–46.63)				
Yes	439 (205)	46.70 (42.03–51.36)	1.23 (0.93–1.63)	0.1450	0.91 (0.65–1.29)	0.607
Gardening or practice of agriculture						
No	488 (202)	41.39 (37.02–45.76)				
Yes	312 (153)	49.04 (43.49–54.59)	1.36 (1.02–1.81)	0.0340	1.15 (0.84–1.58)	0.383
Contact with cats						
No	557 (242)	43.45 (39.33–47.56)				
Yes	243 (113)	46.50 (40.23–52.77)	1.13 (0.83–1.53)	0.424		
Hand washing before eating						
No	214 (122)	57.01 (50.38–63.64)				
Yes	586 (233)	39.76 (35.80–43.72)	0.50 (0.36–0.68)	<0.0001	0.56 (0.40–0.79)	0.001
Marital status						
Single	437 (180)	41.19 (36.58–45.80)				
Married	363 (175)	48.21 (43.07–53.35)	1.33 (1.00–1.76)	0.0470	1.24 (0.82–1.89)	0.304
Occupation						
Students	179 (68)	37.99 (30.88–45.10)				
Other business	223 (112)	50.22 (43.66–56.79)	1.65 (1.10–2.46)	0.0145	0.72 (0.42–1.26)	0.254
Unemployed	163 (62)	38.04 (30.58–45.49)	1.00 (0.65–1.55)	0.9927	0.57 (0.31–1.04)	0.067
Employed	235 (113)	48.09 (41.70–54.47)	1.51 (1.02–2.25)	0.0406	0.74 (0.41–1.33)	0.319

*N*, number tested; OR, odds ratio; CI, confidence interval.

In the univariate analysis, eight variables were identified as possible risk factors associated with *T. gondii* infection: birth place (*p* = 0.0390), age (*p* < 0.0001), residence area (*p* = 0.0280), consumption of undercooked meat (*p* = 0.0130), gardening or practice of agriculture (*p* = 0.0340), eating without washing hands (*p* < 0.0001), marital status (*p* = 0.0470), and occupation (*p* = 0.0190) (Table 1).

Further analysis using multivariate logistic regression identified four variables as potential risk factors of infection among blood donors in Tunisia: birth place (*p* = 0.0340), residence area (*p* = 0.024), age (*p* < 0.0001), and eating without washing hands (*p* = 0.001) (Table 1).

In a second multivariate model including the four variables found to be significant in the first model, we tested interactions between age and birth area, and age and residence area (Table 2). We found a significant interaction between age and birth area (*p* = 0.001) and a borderline interaction between age and residence area (*p* = 0.056). We therefore chose to run multivariate logistic regressions for the four different age categories separately, by including the other three significant variables identified by multivariate logistic regression (birth place, hand washing before eating, and residence area). The results obtained (Table 3) showed that hand washing before eating was not a protective factor in the age category (18–25 years), birth place was not a risk factor in the age category (26–35 years), and residence area was not a risk factor in the age categories (36–45 years) and more than 45 years.

**Table 2:** Best-fitting model multivariate of risk factors for *Toxoplasma gondii* seropositivity.

Risk factors	OR (95% CI)	*p*-value
Region		
Gafsa		
Monastir	2.72 (1.49–4.94)	0.0011
Age (years)		
18–25		
26–35	4.98 (1.50–16.58)	0.0089
36–45	0.44 (0.15–1.30)	0.1363
>45	0.71 (0.21–2.43)	0.5861
Residence area		
Rural		
Urban	0.30 (0.13–0.71)	0.0064
Hand washing before eating		
No		
Yes	0.52 (0.37–0.74)	0.0003
Age (years)*Region		
18–25*Gafsa		
18–25*Monastir		
26–35*Gafsa		
26–35*Monastir	0.26 (0.11–0.57)	0.0009
36–45*Gafsa		
36–45*Monastir	1.14 (0.47–2.80)	0.7714
>45*Gafsa		
>45*Monastir	0.99 (0.33–2.97)	0.9797
Age (years)*Residence area		
18–25*Rural		
18–25*Urban		
26–35*Rural		
26–35*Urban	1.17 (0.32–4.22)	0.8107
36–45*Rural		
36–45*Urban	2.90 (0.87–9.66)	0.0832
>45*Rural		
>45*Urban	5.31 (1.33–21.27)	0.0184

OR, odds ratio; CI, confidence interval.

*: interaction between significant variables.

Hosmer–Lemeshow *χ* ^2^ = 1.50, *p* = 0.959, receiver-operating characteristic curve (ROC) = 0.69.

**Table 3:** Multivariate regression models for *Toxoplasma gondii* seropositivity based on age group.

18–25 years	*N* (positive)	Seroprevalence in % (95% CI)	OR (95% CI)	*p*-value
Region				
Gafsa	100 (25)	25.00 (16.51–33.49)		
Monastir	190 (74)	38.95 (32.01–45.88)	2.50 (1.38–4.52)	0.002
Residence area				
Rural	28 (15)	53.57 (35.10–72.04)		
Urban	262 (84)	32.06 (26.41–37.71)	0.26 (0.11–0.63)	0.003
Hand washing before eating				
No	42 (14)	33.33 (19.08–47.59)		
Yes	248 (85)	34.27 (28.37–40.18)	1.07 (0.50–2.25)	0.857^a^
26–35 years	*N* (positive)	Seroprevalence in % (95% CI)	OR (95% CI)	*p*-value
Region				
Gafsa	132 (82)	62.12 (35.85–70.40)		
Monastir	108 (58)	53.70 (44.30–63.11)	0.65 (0.37–1.13)	0.132^a^
Residence area				
Rural	31 (25)	80.65 (66.74–94.55)		
Urban	209 (115)	55.02 (48.28–61.77)	0.36 (0.14–0.95)	0.039
Hand washing before eating				
No	75 (55)	73.33 (63.33–83.34)		
Yes	165 (85)	51.52 (43.89–59.14)	0.38 (0.20–0.70)	0.002
36–45 years	*N* (positive)	Seroprevalence in % (95% CI)	OR (95% CI)	*p*-value
Region				
Gafsa	108 (28)	25.93 (17.66–34.19)		
Monastir	67 (35)	52.24 (40.28–64.20)	3.03 (1.58–5.82)	0.001
Residence area				
Rural	34 (12)	35.29 (19.23–51.36)		
Urban	141 (51)	36.17 (28.24–44.10)	0.86 (0.37–2.01)	0.746^a^
Hand washing before eating				
No	64 (30)	46.88 (34.65–59.10)		
Yes	111 (33)	29.73 (21.23–38.23)	0.50 (0.26–0.97)	0.042
More than 45 years	*N* (positive)	Seroprevalence in % (95% CI)	OR (95% CI)	*p*-value
Region				
Gafsa	60 (28)	46.67 (34.04–59.29)		
Monastir	35 (25)	71.43 (56.46–86.40)	3.02 (1.20–7.54)	0.018
Residence area				
Rural	18 (8)	44.44 (21.49–67.40)		
Urban	77 (45)	58.44 (47.43–69.45)	1.83 (0.58–5.80)	0.299^a^
Hand washing before eating				
No	33 (23)	69.70 (54.02–85.38)		
Yes	62 (30)	48.39 (35.95–60.83)	0.38 (0.15–0.96)	0.041

a Non-significant results.

## Discussion

This is the first sero-epidemiologic study of *T. gondii* infection among healthy blood donors who attended two blood banks in South-West and Central-East Tunisia. In this study, we found an overall seroprevalence of *T. gondii* antibodies of 44.4% (95% CI: 40.93–47.82). Furthermore, patients with both IgM and IgG antibodies had a high avidity for IgG, suggesting previous infection of more than four months, which poses no threat to blood recipients.

Our overall seroprevalence finding is consistent with that found in Tunis, North Tunisia, (47.7%) in a cross-sectional study including all pregnant women who presented for serological testing of toxoplasmosis [11]. A retrospective study in the Sfax region, East Tunisia, showed that the seroprevalence rate of pregnant women was 46.1% during the years 1994–1997, 41.3% from 1998 to 2001, and 36.9% from 2002 to 2006 [34]. An earlier study showed that the seroprevalence in the general population from Tunisia was 45.9% [3].

In the Maghreb region (North Africa), few studies have been carried out on the prevalence of *Toxoplasma* antibodies in blood donors and the general population. Most studies focused on the prevalence of *T. gondii* in pregnant women, and showed a seroprevalence rate similar to ours due to shared sociocultural habits: 44.8% in Libya [28], 47.8% in Algeria [26], and 51% in Morocco [22].

Compared to other African countries, our overall seroprevalence was lower than that reported in West Africa [35], Ethiopia [16] and Egypt [9], where seroprevalence of more than 59% has been reported in healthy blood donors or the general population. However, some of these countries have significant risk factors that are worth mentioning, such as the habit of consuming raw beef in Ethiopia [30].

The seroprevalence found in donors from Coastal Tunisia (48.0%) is comparable with the seroprevalence of *T. gondii* infection reported in the control group of blood donors (53.8%) during a comparative study between schizophrenic patients and healthy controls [10] in Tunisia. Similar results were found in the general population of Northern Tunisia (58.4%) [4] and in pregnant women who consulted the parasitology laboratory of the Pasteur Institute of Tunis (45.6%) [1]. In our study, seroprevalence found in South Tunisia (40.7%) was significantly lower than that found in blood donors at the coast (*p* < 0.05).

The variation in the seroprevalence of *T. gondii* infection among the blood donors may be the result of differences in the geographic and environmental characteristics of blood donors in each region (adjusted OR = 2.72; 95% CI: 1.49–4.94; *p* < 0.0011). The city of Gafsa has a hot desert climate and the seroprevalence of *T. gondii* infection is usually lower in dry climates than in humid ones [6]. In the regions that have milder temperatures and higher humidity rates, like the Monastir region, the chances of survival of oocysts in the environment are higher [31] and therefore the rate of transmission of *T. gondii* by contaminated soil or food is higher.

In this context, we should not neglect the maritime domain since a seroprevalence among fishermen of 80% was found; this seems to be an additional source of contamination for the coastal region of Monastir. High levels of *T. gondii* has been demonstrated in some aquatic animals [24]; the consumption of contaminated mussels [17] could represent an additional risk factor that should be taken into consideration. Previous studies indicated that *T. gondii* oocysts may be transported by the flow of freshwater into the ocean [6], which represents a source of contamination of mussels [7], and this may partly explain the high prevalence of *T. gondii* infections in coastal provinces.

In addition, our results show that participants living in urban areas (42.8%) are less infected (adjusted OR = 0.30; 95% CI: 0.13–0.71) than those living in rural areas (54.05%), where contamination of the environment by *T. gondii* oocysts and exposure to the parasite are higher. These findings are consistent with the study of Tammam et al. [36] conducted in Qena governorate of Egypt, which showed a higher seroprevalence among women living in rural areas than in the urban areas [36]. This significant difference could be attributed to occupational activities linked to contact with soil or animals, and to living in lower socio-economic and lower hygiene lifestyle levels [15, 22]. However, according to a study carried out in North Tunisia and in Tanzania, a higher seroprevalence of toxoplasmosis was found in urban than in rural areas [4, 29].

Irrespective of the residence area, it is known that hand contamination plays an important role in the transmission of germs and pathogenic parasites. In this context, hand hygiene was taken into account in this study. Hand washing before eating was identified as a protective factor of *T. gondii* seropositivity (adjusted OR = 0.52; 95% CI: 0.37–0.74). Thus, *T. gondii* infection can be acquired by ingestion of oocysts; a similar finding had previously been reported by several studies showing that the risk of exposure to *Toxoplasma* increases by neglecting hand washing after handling cat litter [2], as well as having contact with the ground, particularly in rural communities [8, 15].

In this context, a survey carried out in the Sousse region (North-East Tunisia) on the occasion of the Muslim sacrifice feast, showed also that the majority of meat handlers did not respect the hygiene rules, since 91% of them did not wash their hands after handling meat and before eating [21].

Multivariate logistic regression analysis shows that seropositivity to *T. gondii* was strongly associated with the age of the donors. In fact, the age range (26–35 years) was five times more likely to be seropositive for *T. gondii* compared to older age group (>45 years), which seems to reflect the acquisition of a new lifestyle by the younger age group that increases contact with the parasite. In the same context, a study carried out in Abidjan, Côte d’Ivoire, proved that seroprevalence decreases with increasing age [35]. However, several studies have shown that the infection rate increases with age, due to high exposure to sources of infection [4, 8, 12, 28]. Contrarily, research performed by Fakhfakh and Elsheikha did not find any correlation between the presence of *T. gondii* antibodies and age [9, 11].

Concerning the significant variables of the final model (place of birth, washing hands before eating, and place of residence), we separately tested the four different age categories (Table 3). The analysis showed that hand washing is not protective in the age group (18–25), which may be explained by the fact that young people are frequently away from their homes either to work or to study, thus increasing the risk of infection (risky occupation, eating outdoor) or by ignorance of good hand washing (washing frequency and manner).

The multivariate logistic regressions also showed that the region (birth place) is not a risk factor in the age group (26–35). Being the third largest industrial city and having the second largest universities of Tunisia, the Monastir region attracts massive inflows of students and workers. However, the region of Gafsa has substantial departure towards the regions of northern Tunisia and the Sahel where factories and universities are concentrated.

We have also noted that the place of residence (urban/rural) is not a risk factor for donors (36–45 years old) and for people (>45 years old). In fact, this age group, in rural or in urban areas, is not subject to acquiring the disease. This age group is generally married so they eat at home and hygiene measures are taken before, during or after meals thanks to awareness programs implemented by public health teams.

In conclusion, this population-based investigation adds valuable contributions to the understanding of risk factors for *T. gondii* infection in Tunisia. Age and birth place have been identified as potential risk factors for acquiring infection in blood donors. Hand washing before eating and living in urban areas have been found to be protective factors. On the other hand, blood transfusion does not seem to be a risk factor of transmission, given the low seroprevalence of anti-*Toxoplasma* IgM antibodies in blood donors. Efficient educational and environmental intervention strategies are also required to reduce the risk of *T. gondii* transmission by raising awareness about the importance of hand washing, and by promoting public policy measures aimed at improving sanitation.

## Conflict of interest

Authors declared no conflict of interests.
